# Therapeutic Effects of GLP-1 Receptor Agonists and DPP-4 Inhibitors in Neuropathic Pain: Mechanisms and Clinical Implications

**DOI:** 10.3390/biom15050622

**Published:** 2025-04-26

**Authors:** Yaswanth Kuthati, Venkata Naga Goutham Davuluri, Chih-Shung Wong

**Affiliations:** 1 Department of Anesthesiology, Cathay General Hospital, Taipei 10630, Taiwan; yaswanthk1987@gmail.com; 2 Department of Pathology and Immunology, Baylor College of Medicine, Houston, TX 77030, USA; goutibiotech76@gmail.com; 3 Graduate Institute of Medical Science, National Defense Medical Center, Taipei 11467, Taiwan

**Keywords:** glucagon-like peptide-1, dipeptidyl-peptidase-IV inhibitors, neuropathic pain, oxidative stress, inflammation

## Abstract

Glucagon-like peptide-1 (GLP-1) is a peptide hormone secreted by the small intestine upon food intake. GLP-1 enhances insulin secretion, suppresses glucagon release, and promotes satiety, resulting in reduced food consumption and subsequent weight loss. Endogenous GLP-1 has a very short half-life and is rapidly degraded by the enzyme dipeptidyl-peptidase-IV (DPP-4). To address this limitation, GLP-1 receptor agonists (GLP-1RAs) and DPP-4 inhibitors (DPP-4is) were developed and have demonstrated potency in clinical practice. In recent years, GLP-1RA and DPP4-i therapies are known to have pleiotropic effects, such as a reduction in oxidative stress, autophagy regulation, metabolic reprogramming, enhancement of anti-inflammatory signaling, regulation of gene expression, and being neuroprotective. These effects imply a therapeutic perspective for GLP-1RA and DPP-4i therapies in neuropathic pain treatment. Preclinical and clinical studies increasingly support the hypothesis that these therapies may alleviate neuropathic pain by targeting multiple mechanisms that induce neuropathic pain, such as inflammation, oxidative stress, and mitochondrial dysfunction. This review explores the mechanisms by which GLP-1RAs and DPP-4is alleviate neuropathic pain. It also highlights current advancements in incretin research, focusing on the therapeutic effects of GLP-1RAs and DPP-4-is for neuropathic pain.

## 1. Introduction

Neuropathic pain is a condition that occurs following a primary lesion or disease impacting the somatosensory nervous system. It is characterized by heightened sensitivity to pain (hyperalgesia) or diminished sensitivity (hypoalgesia). Neuropathic pain affects about 7–10% of the population worldwide, greatly affecting the quality of life and becoming an economic burden [1]. The common causes for this condition include diabetes, postherpetic neuralgia, treatment with chemotherapeutic drugs, and nerve injury resulting from surgery or accidents [2]. The pathophysiology of neuropathic pain encompasses multiple factors, including both peripheral and central sensitization [3]. The main mechanisms comprise neuroinflammation and oxidative stress, leading to sustained neuronal hyperexcitability and the persistence of chronic pain [4]. Pro-inflammatory cytokines, activation of glial cells, and high levels of reactive oxygen species production (ROS) are known to increase nerve damage and trigger pain signaling pathways [5].

Recently, a lot of attention has been paid to how glucagon-like peptide-1 (GLP-1) and its receptor (GLP-1R) signaling affect neuropathic pain. Multiple chronic pain models have evidenced the impacts of GLP-1 receptor agonists (GLP-1Ras) and their analogs [6,7]. GLP-1, an incretin hormone, is released by enteroendocrine L-cells in the distal jejunum, ileum, and colon, where it interacts with the GLP-1R [8]. GLP-1 plays a crucial role in stimulating insulin release from pancreatic islets in response to elevated glucose levels following food consumption [8].

DPP-4 inhibitors (DPP-4is) are antidiabetic agents approved for the management of type 2 diabetes [9]. They primarily function by blocking the enzyme DPP-4, which degrades GLP-1. DPP-4i extends the half-life of GLP-1, thereby enhancing insulin production and diminishing glucagon release, which leads to improved glycemic control in individuals with type 2 diabetes mellitus (T2DM) [9]. Apart from metabolic effects, GLP-1 and GLP-1R were recently found to be involved in neuroprotection and anti-inflammation [9]. According to some preclinical reports, GLP-1R activation is known to control pain pathways, in particular neuropathic pain, through a reduction in neuroinflammation, the alleviation of oxidative stress, and the hyperexcitation of the neurons [9]. Since DPP4-i can indirectly enhance GLP-1 circulation, there is great interest in its potential for the management of neuropathic pain.

Preclinical studies indicate that DPP4-is may alleviate pain-related behaviors in models of nerve injury and diabetes-induced neuropathy, perhaps through mechanisms such as the suppression of pro-inflammatory cytokines, inhibition of glial cell activation, and enhancement of neuronal survival [10]. Our research on DPP4-is and GLP-1RAs further investigated these mechanisms and their potential therapeutic benefits in neuropathic pain management [11,12,13]. Nonetheless, despite these encouraging results, further study, including clinical trials, is required to determine the efficacy, tolerability, and underlying mechanisms of DPP4-is in the treatment of neuropathic pain. If verified, these pharmaceuticals may provide an innovative therapeutic strategy for individuals with persistent neuropathic pain, especially those with diabetes-related neuropathy.

## 2. Literature Search on GLP-1RA-Based Therapies for Neuropathic Pain

Recent studies have demonstrated the efficacy of glucagon-like peptide-1 receptor agonists (GLP-1RAs) in managing neuropathic pain, commonly linked to diabetic neuropathy and various chronic pain disorders. GLP-1 receptor agonists, including liraglutide and semaglutide, demonstrate neuroprotective effects by modulating neuroinflammation and pathways related to neuronal survival [14]. Research utilizing animal models indicates that GLP-1 receptor agonists (GLP-1RAs) may alleviate pain hypersensitivity, potentially through the activation of GLP-1 receptors in the central nervous system [15]. The findings indicate a potential new approach for treating neuropathic pain, especially in diabetic neuropathy, where traditional therapies frequently offer insufficient relief.

The efficacy of GLP-1 receptor agonists (GLP-1RAs) in pain modulation is primarily attributed to their anti-inflammatory properties [16]. In rodent models of diabetic neuropathy, GLP-1 receptor agonists have demonstrated a reduction in oxidative stress and inhibition of pro-inflammatory cytokines, both of which are implicated in neuropathic pain [16]. Furthermore, GLP-1RAs may facilitate the regeneration of peripheral nerves, providing a dual therapeutic effect of pain relief and nerve and organ repair [17].

Preliminary evidence from clinical trials in humans supports the effectiveness of GLP-1 receptor agonists in managing neuropathic pain. Some clinical evidence in a small number of patients indicated a significant reduction in peripheral neuropathy in patients with diabetic neuropathy treated with GLP1RAs [18]. Further studies are necessary to determine optimal dosing regimens, long-term safety, and the precise mechanisms through which GLP-1RAs produce these analgesic effects.

The combination of GLP-1RAs with other therapies, including anticonvulsants or antidepressants, may provide improved pain relief, despite the promising results observed. Combination therapies may effectively address the multifactorial nature of neuropathic pain by targeting multiple pain pathways concurrently [19,20]. Clinical trials examining these combinations are currently limited, necessitating further investigation to assess their clinical feasibility and benefits.

## 3. Methods

### 3.1. Literature Search Strategy

We searched the following electronic databases: PubMed, Scopus, and Web of Science. The search was conducted in March 2025, and we checked papers published from January 2000 to March 2025. The keywords we used were as follows:

(“GLP-1 receptor agonists” OR “DPP-4 inhibitors”) AND
(“neuropathic pain”) “Mechanisms” AND (“neuropathic pain” OR “pain management”)

### 3.2. Inclusion and Exclusion Criteria

We included the original research articles or reviews that examined the therapeutic effects of GLP-1 receptor agonists or DPP-4 inhibitors on neuropathic pain. Only English-language publications were included in our study, and papers published in peer-reviewed journals were included. We did not include any case reports, abstracts without full texts, and works that did not focus on the relevant therapeutic agents or pain models.

### 3.3. Data Synthesis and Categorization

A thematic synthesis approach was employed to categorize the findings based on the mechanisms of action, therapeutic efficacy, and clinical outcomes. Articles were grouped by therapeutic agent and neuropathic pain model, with the key findings summarized in tables and discussed qualitatively.

## 4. Pathophysiology of Chronic Pain

Chronic pain, particularly neuropathic pain, occurs from continuous injury or damage to the somatosensory nervous system. Typical symptoms include unusual pain signals and increased pain sensitivity. Neuroinflammation, oxidative stress, and mitochondrial dysfunction are the major pathways involved in the pathophysiology and maintenance of chronic pain syndromes.

### 4.1. Neuroinflammation

Neuroinflammation plays a key role in the onset and maintenance of neuropathic pain. After a nerve injury, immune cells, including microglia and astrocytes, are activated within the central nervous system (CNS) [21]. The microglial cells secrete a series of pro-inflammatory cytokines, such as interleukin-1β (*IL-1β*), tumor necrosis factor-alpha (*TNF-α),* and interleukin-6 (*IL-6*), which heighten neuronal sensitivity and impair standard nociceptive processing [22]. The inflammatory conditions activate pain signaling by enhancing excitatory neurotransmission and inhibiting inhibitory pathways [23]. Prolonged activation of microglial cells will result in central sensitization, a characteristic of chronic pain, in which the CNS becomes hypersensitive.

### 4.2. Oxidative Stress

Oxidative stress, characterized by an imbalance between ROS generation and antioxidant defense mechanisms, plays a major role in neuronal damage and pain hypersensitivity [24]. An increase in ROS production leads to lipid peroxidation, DNA damage, and protein malfunction, affecting the normal function of neurons [24,25]. During neuropathic pain, oxidative stress aggravates inflammation and directly affects the function of ion channels, thereby enhancing pain transmission [26]. The hallmarks of oxidative stress, including malondialdehyde (MDA) and 8-hydroxy-2′-deoxyguanosine (8-OHdG), are often elevated in chronic pain animal models, highlighting the pathophysiological significance of oxidative damage [26].

### 4.3. Mitochondrial Impairment

Mitochondria play a vital role in cellular energy metabolism and calcium regulation. A failure of mitochondrial function in chronic pain conditions is known to reduce ATP production with the upregulation of intracellular calcium levels and increase ROS formation, all of which lead to neuronal degeneration and limit axonal transport [27]. This disruption, including excessive fragmentation and diminished biogenesis, is observed in chronic pain animal models [27]. Abnormal mitochondrial function undermines neuronal integrity and initiates pro-apoptotic pathways, thereby exacerbating pain through ongoing neurodegeneration [27]. The restoration of mitochondrial function has become a viable approach for alleviating chronic pain and enhancing neuronal survival.

Neuroinflammation, oxidative stress, and mitochondrial dysfunction collectively prolong and intensify pain signaling pathways in chronic pain conditions. Understanding these interconnected pathways is essential for the production of new pharmaceutical therapies that can fix the faulty feedback loops sustaining chronic pain.

## 5. Physiological and Pathophysiological Roles of GLP-1 and DPP-4 in the Nervous System

GLP-1 and DPP-4 are known to regulate glucose levels in the body, though emerging data highlight their significant role in the nervous system [28]. GLP-1, via its receptor GLP-1R, is present in multiple brain areas, such as the hypothalamus, brainstem, and hippocampus, where it influences neuroprotection, synaptic plasticity, hunger, and cognition [29].

DPP-4, an enzyme that swiftly destroys GLP-1, is also present in brain tissue and affects neuroimmune interactions [30]. Inhibition of DPP-4 extends GLP-1 activity and independently influences neuronal signaling and inflammation [31]. The GLP-1/DPP-4 axis is a promising target for neurological disorders such as Alzheimer’s disease, Parkinson’s disease, and neuropathic pain [32].

Autoradiography of human and animal brain sections has revealed a high expression of GLP-1R, highlighting its significant role in the central nervous system (CNS) [33,34]. More specifically, GLP-1R is distributed across various brain regions, including the hippocampus, septal nucleus, neocortex, brainstem, cerebellum, and hypothalamus [35,36,37]. Although GLP-1R is profoundly found in neurons, a recent study involving mRNA analysis showed its expression in astrocytes and microglia in the cerebral cortex [38]. This widespread expression underscores its involvement in regulating appetite, energy balance, and potentially cognitive functions. Notably, currently approved GLP-1RAs such as semaglutide, exenatide, dulaglutide, and liraglutide are known to cross the blood–brain barrier (BBB), thereby gaining access to the CNS [39,40].

Neuronal expression of GLP-1RAs is important for weight loss actions, signifying that these receptors mediate satiety and suppress food intake through appetite-suppressing circuits in the hypothalamus and other areas [30,35,36]. This also demonstrates anti-inflammatory and anti-apoptotic properties, which are essential in neurodegenerative disorders [35,41,42].

However, the expression of GLP-1R in the spinal cord remains a topic of debate. Vahl and colleagues analyzed multiple tissues of Sprague–Dawley rats and reported undetectable expression levels of GLP-1R mRNA in the spinal cord [37]. Contrastingly, other studies have demonstrated the presence of GLP-1R expression in the spinal cord. For instance, Li et al. reported the expression of GLP-1R in neuroblastoma cells and spinal cord sections of mice, supporting the essentiality of GLP-1R-mediated effects in this region [43]. Additionally, the CNS expresses GLP-1R and has been associated with a range of neuroprotective effects, including the presence of oxidative stress, neurogenesis improvement and modulation of neuroinflammatory pathways [44]. These results imply that GLP-1R-targeted therapies could be potentially used to treat neurodegenerative diseases, including diseases like Alzheimer’s and Parkinson’s as well as neuropathic pain and cancer [10,45]. GLP-1RAs may exert their therapeutic effects on neuropathic pain through mechanisms within the CNS, in addition to peripheral actions. However, further work is needed to reconcile the disagreements concerning spinal cord expression and better define the therapeutic implications.

## 6. Neuroprotective Benefits of GLP-1R Agonists and DPP-4 Inhibitors: Anti-Inflammatory and Antioxidant Mechanisms

### 6.1. DPP-4is

DPP-4is are low-molecular-weight drugs taken orally that were introduced in 2006 for the treatment of type 2 diabetes mellitus [46]. Dipeptidyl-peptidase-IV (DPP-4) is an enzyme located on epithelial and endothelial cells, expressed in several tissues throughout the body, including the lungs, liver, kidneys, small intestine, brain, and spinal cord [47,48]. DPP-4 deactivates incretin hormones such as glucagon-like peptide-1 (GLP-1) and gastric inhibitory peptide (GIP), producing inactive metabolites GLP-1(9–36) amide and GIP (3–42) [49].

DPP-4is selectively inhibit DPP-4 activity, leading to a two-to-threefold increase in intrinsic GLP-1 and GIP levels following food intake [9]. Currently approved DPP-4is for T2DM include teneligliptin, alogliptin, linagliptin, saxagliptin, sitagliptin, and vildagliptin. All these drugs demonstrate comparable glycemic effects, with modest reductions in Hemoglobin A1c (HbA1c) levels ranging from 0.5% to 0.8% [50]. Unlike GLP-1RAs, DPP-4is have no significant impact on body weight due to the limited enhancement of GLP-1 activity [51,52,53]. Moreover, they are associated with a very low risk of hypoglycemia, owing to their glucose-dependent mechanism of action mediated by GLP-1 [49].

### 6.2. GLP-1RAs

GLP-1RAs enhance glucose-dependent insulin secretion, suppresses glucagon release during hyperglycemia, slow gastric emptying, prevent sharp increases in postprandial glucose, and reduce food intake, leading to weight loss [54,55,56,57]. These medications are classified into two groups based on their duration of receptor activation.

### 6.3. Short-Acting GLP-1RAs

Short-acting agents, such as exenatide (administered twice daily) and lixisenatide (once daily), provide transient receptor activation [58]. They are particularly effective in reducing postprandial glucose levels by delaying gastric emptying but have minimal impact on fasting plasma glucose (FPG) or nocturnal glucose levels [58]. These agents maintain their effect on gastric motility during long-term use [58].

### 6.4. Long-Acting GLP-1RAs

Long-acting agents, including semaglutide (weekly), extended-release exenatide, albiglutide, dulaglutide, and taspoglutide, provide sustained receptor activation [58]. These drugs effectively reduce nocturnal and fasting plasma glucose levels and significantly lower HbA1c [58].

In terms of glycemic control, GLP-1RAs reduce HbA1c by approximately 1.1% to 1.6%, compared to reductions of 0.6% to 1.1% observed with DPP-4is [59]. Due to their strong glycemic effects, weight loss benefits, and low risk of hypoglycemia, GLP-1RAs are often recommended as the first injectable glucose-lowering therapy for patients with type T2DM, even before initiating insulin in many cases [60]. All GLP-1 RAs are administered through subcutaneous injection, except for oral semaglutide, with the most common adverse effects being gastrointestinal issues (nausea, vomiting, diarrhea) and injection site reactions [61].

Emerging evidence highlights the potential of GLP-1RAs and DPP-4is in alleviating neuropathic pain by targeting inflammation, oxidative stress, and mitochondrial dysfunction [62]. These therapies modulate key pathways involved in pain signaling, providing a novel approach to neuropathic pain treatment. Preclinical studies show promise, and clinical trials are ongoing to validate their efficacy in neuropathic pain management.

### 6.5. Targeting Inflammation in Neuropathic Pain

Neuropathic pain is often associated with chronic inflammation in both the peripheral and central nervous systems [63]. Several studies have indicated that GLP-1RAs and DPP-4is can modulate inflammatory pathways, which may contribute to their analgesic effects [64,65]. By reducing the expression of pro-inflammatory cytokines and activating anti-inflammatory signaling, GLP-1RA-based therapies may mitigate the inflammatory processes that sustain neuropathic pain [66,67,68]. GLP-1RAs such as liraglutide and exenatide decrease pro-inflammatory cytokines, tumor necrosis factor-alpha (*TNF-α*), and interleukin-1β (IL-18) in rodent mouse models of diabetic neuropathy [69,70].

These cytokines contribute to the sensitization of nociceptors, leading to neuropathic pain [24,35]. Furthermore, these therapies have been shown to reduce the activation of microglial cells, key immune cells that play a significant role in triggering chronic pain in the central nervous system [36]. Additionally, activation of GLP-1R has previously been demonstrated to induce *IL-10* expression in chronic pain models [23], supporting the decisive role of GLP-1R in mediating the resolution of inflammation. *IL-10* acts as a major inhibitor of proinflammatory cytokines, such as *IL-2*, *TNF-α*, and *CCL2*, while promoting anti-inflammatory factors like *TGF-β* and nerve growth factors [23]. These functions highlight the crucial role that *IL-10* plays in the protection of the host from immune-mediated damage and its action as a pain-modulating cytokine in acute inflammatory and neuropathic pain models.

**Figure 1:**
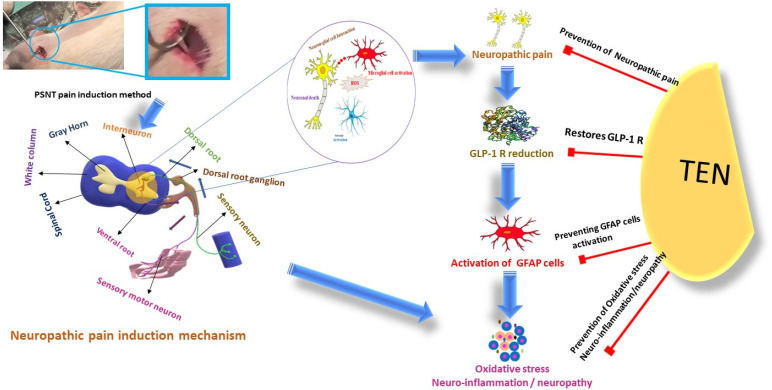
Mechanism of neuropathic pain induction and the potential role of teneligliptin.

Emerging evidence suggests that GLP-1RAs and DPP-4is, such as Teneligliptin, hold significant potential in the management of neuropathic pain by targeting inflammation, oxidative stress, and mitochondrial dysfunction [70]. Figure 1 in this review illustrates the therapeutic role of Teneligliptin, a DPP-4i, in alleviating neuropathic pain, with a focus on its modulation of inflammatory processes [16]. Chronic inflammation is a hallmark of neuropathic pain, with nerve-injury-induced activation of glial cells in the central nervous system leading to the release of pro-inflammatory cytokines such as *TNF-α*, *IL-6*, and *IL-1β*, which contribute to pain hypersensitivity and persistent pain [16,71]. This work highlights that Teneligliptin, through inhibition of DPP-4, prolongs the activity of GLP-1. By enhancing GLP-1 signaling, Teneligliptin helps reduce the expression of pro-inflammatory cytokines, thereby decreasing both peripheral and central nervous system inflammation [16]. One of the key findings of our work is that Teneligliptin may also reduce the activation of glial cells, which are central mediators of neuroinflammation in neuropathic pain [16]. By inhibiting glial cell activation, Teneligliptin prevents the release of pro-inflammatory mediators that sensitize pain pathways, offering a mechanism for reducing neuroinflammation and the alleviation of neuropathic pain [16]. Furthermore, oxidative stress, which contributes to cellular damage and pain signaling, is another crucial factor in neuropathic pain. Our research suggests that Teneligliptin, through GLP-1R activation, possesses antioxidant properties that reduce reactive oxygen species (ROS) levels, thus protecting neural tissues from oxidative damage and mitigating the severity of pain [16]. Additionally, Teneligliptin may offer neuroprotective effects by restoring mitochondrial function, which is often impaired in neuropathic pain [72]. This mitochondrial dysfunction contributes to both cellular injury and exacerbated pain signaling [73]. Preclinical studies from our research indicate that Teneligliptin significantly reduces pain hypersensitivity in animal models of neuropathic pain, supporting its potential to address both the inflammatory and oxidative components of neuropathic pain [11]. Models of neuropathic pain using animals have demonstrated that GLP-1RAs have the capacity to diminish the production of pro-inflammatory cytokines such as *TNF*-α and *IL-1β*, which are elevated in chronic pain conditions [74,75]. For instance, liraglutide (GLP-1RA) administration in rats suffering from diabetic neuropathy reduced inflammatory markers and improved pain behaviors [76]. Research studies on diabetic neuropathy models indicated that glucagon-like peptide-1 receptor agonists, including exenatide, are known to decrease markers of oxidative stress in spinal cord tissues [77]. In particular, treatment with GLP-1RAs reduced levels of malondialdehyde, a product of lipid peroxidation [78].

As clinical trials progress, the findings from our work suggest that Teneligliptin may provide a novel, safer alternative to traditional pain management strategies, particularly those reliant on opioids or NSAIDs, which are associated with adverse side effects [12]. If validated in clinical settings, Teneligliptin could become an important therapeutic option for patients suffering from neuropathic pain, offering relief not only from pain symptoms but also from the underlying pathophysiological mechanisms driving neuropathic pain.

In addition to its role in neuropathic pain, Teneligliptin may also play a crucial role in mitigating morphine tolerance, a significant challenge in chronic pain management. Long-term opioid therapy often leads to reduced analgesic efficacy, requiring higher doses that increase the risk of dependency and adverse effects [79]. Our pre-clinical research suggests that Teneligliptin, through its anti-inflammatory and antioxidant mechanisms, may attenuate opioid-induced glial cell activation, which is a key driver of morphine tolerance [12]. By inhibiting DPP-4 and enhancing GLP-1 signaling, Teneligliptin can suppress neuroinflammation, reduce oxidative stress, and potentially restore opioid responsiveness [12]. Our study indicates that co-administration of Teneligliptin with morphine may help maintain opioid efficacy while reducing dose escalation, thereby offering a promising adjunct therapy for chronic pain patients requiring long-term opioid treatment [12]. Future investigations into the combined use of Teneligliptin and opioids could further elucidate its potential in improving pain management strategies while minimizing opioid-related risks.

In humans, DPP-4is, including sitagliptin, have demonstrated the ability to decrease systemic inflammatory mediators, including C-reactive protein (CRP) and interleukin-6 (IL-6) and other inflammatory markers in human patients with T2DM in clinical trials [80,81]. While direct evidence in neuropathic pain patients is limited, the anti-inflammatory effects of GLP-1RA-based therapies could play a significant role in managing pain.

In humans, DPP-4is, including sitagliptin, have demonstrated a significant ability to decrease systemic inflammatory mediators such as CRP and IL-6 in patients with T2DM [82]. These findings have been consistently observed in multiple clinical trials, where the use of DPP-4is resulted in a notable reduction in inflammatory biomarkers [82]. CRP and IL-6 are key indicators of systemic inflammation and have been linked to various chronic conditions, including cardiovascular disease and insulin resistance, both of which are commonly seen in individuals with T2DM [83].

### 6.6. Modulation of Oxidative Stress and Central Mechanisms in Neuropathic Pain: Molecular Pathways, Mitochondrial Protection, and Clinical Implications

Oxidative stress, which results from an imbalance between ROS and the body’s antioxidant defenses, is a key mechanism in the pathogenesis of neuropathic pain [84]. It also plays a critical role in the development and maintenance of neuropathic pain by causing cellular damage and impairing neuronal function [85]. GLP-1RA-based therapies are believed to exert antioxidant effects, protecting cells from damage and alleviating pain. GLP-1-RA-based therapies, through their antioxidant properties, may help alleviate oxidative stress, reduce neuronal damage, and, consequently, decrease pain [86]. Activation of GLP-1R has been associated with the induction of antioxidant enzymes, such as superoxide dismutase (*SOD*) and catalase, which are involved in the neutralization of ROS [87]. This decrease in ROS assists in neuroprotection from oxidative stress damage, characteristic of neuropathic pain diseases like diabetic neuropathy and chemotherapy-induced peripheral neuropathy [88,89,90,91]. 

GLP-1RAs have been shown to lower the oxidative stress markers malondialdehyde (MDA) and 8-hydroxy-2′-deoxyguanosine (8-OHdG; byproducts of oxidative damage) in animal models of neuropathy [80]. Additionally, GLP-1-RA-based treatments have been demonstrated to protect against ROS-induced mitochondrial dysfunction, a common feature of neuropathic pain progression [92]. While the clinical evidence specifically targeting oxidative stress in neuropathic pain is still limited, the broader benefits of GLP-1RAs in reducing oxidative stress have been observed in conditions such as cardiovascular disease and diabetes [93]. These findings support the potential of GLP-1-RA-based therapies in reducing oxidative damage in neuropathic pain.

Mitochondrial dysfunction is a key contributor to the pathophysiology of neuropathic pain. Impaired mitochondrial function can lead to energy deficits, altered axonal transport, and increased neuronal damage [94,95]. GLP-1-RA-based therapies may help restore mitochondrial function, thereby alleviating pain and promoting neuronal survival.

GLP-1RAs (including liraglutide and exenatide) prove to be beneficial in improving mitochondrial dynamics, increasing mitochondrial biogenesis, and preventing mitochondrial fragmentation [96]. Consequently, enhanced ATP generation and inhibition of neuronal apoptosis are achieved. GLP-1RAs have been shown to restore mitochondrial function in peripheral nerves and reduce nerve fiber degeneration in animal models of neuropathic pain [92].

The beneficial effects of GLP-1RAs on mitochondrial function may be mediated via the activation of pathways involved in the regulation of mitochondrial function, including the AMP-activated protein kinase (AMPK) pathway (a major regulator of mitochondrial biogenesis) and the peroxisome-proliferator-activated receptor gamma coactivator 1-alpha (PGC-1α) [97,98]. These pathways are activated by GLP-1RA-based therapies, which help preserve neuronal integrity and prevent neuropathic-pain-associated degeneration [42,43]. Given the role of mitochondrial dysfunction in neuropathic pain, the mitochondrial-protective effects of GLP-1RAs offer a promising therapeutic strategy. Restoring mitochondrial health may not only reduce pain but also promote nerve regeneration and repair, addressing both the symptoms and the underlying causes of neuropathic pain. Preclinical studies have demonstrated that GLP-1RAs can restore mitochondrial integrity and improve the energy production of neurons [99]. For example, liraglutide treatment in diabetic rats improved mitochondrial function and reduced nerve fiber degeneration in the sciatic nerve [76].

The CNS plays a critical role in the development and modulation of neuropathic pain [91]. GLP-1Rs are expressed in several regions of the brain and spinal cord that are involved in pain perception and regulation [87]. There is an ongoing debate concerning the role of GLP-1R in the spinal cord; however, some reports indicate that these receptors may play a direct role in pain processing from spinal cord neurons [6]. For example, using neuropathic pain models, administration of GLP-1RAs such as liraglutide resulted in altered pain perception and less pain sensitivity, probably through spinal pain pathway modulation [100].

Activation of GLP-1RAs in these regions may modulate pain signaling, providing another potential mechanism for the analgesic effects of GLP-1-RA-based therapies. The central processing of pain is closely regulated by various neuropeptides, including GLP-1. GLP-1RAs may modulate pain transmission in the CNS by interacting with pain pathways in the brain and spinal cord [101]. GLP-1RAs such as semaglutide are known to cross the blood–brain barrier, suggesting that their pain-relieving effects could be mediated through brain structures such as the hypothalamus and brainstem, areas that regulate nociception [102]. Clinical evidence for this mechanism is under development, but the interface between central pain processing systems and GLP-1RAs provides an intriguing target in the treatment of neuropathic pain.

Beyond neuropathic pain, the neuroprotective abilities of GLP-1RAs have also been explored in Alzheimer’s and Parkinson’s diseases, characterized by their combination of mitochondrial dysfunction and neurodegeneration [99]. One recent study showed that exenatide was capable of improving mitochondrial bioenergetics and reducing amyloid plaque accumulation in an animal model for Alzheimer’s disease [99].

The importance of GLP-1Rs in the spinal cord is still being debated, but recent studies have indicated that the expression of these receptors in the spinal cord may affect the processing of pain in nerve cells of the spinal cord [6,103]. GLP-1RAs have been demonstrated to modulate nociceptive transmission mediated by spinal cord neurons that transmit peripheral pain signals to the brain [15]. In addition, brainstem and hypothalamic GLP-1R activation, which are well established locations of pain modulation, could further contribute to the analgesic effects of these therapies [87]. Indeed, there exist limited studies directly evaluating the effect of GLP-1RAs on central pain, but modulation of the central pain pathways by GLP-1-RA-based therapies has been proposed in patient groups suffering from fibromyalgia and chronic lower back pain [100]. In a study involving diabetes patients, GLP-1RAs reduced pain scores and improved mood, suggesting a potential role for central pain modulation [104]. One of the key features of neuropathic pain is central sensitization, where the CNS becomes hyper-responsive to pain stimuli [105]. By modulating central pain processing and reducing neuroinflammation, GLP-1RAs may help prevent or reverse central sensitization, offering a new approach for managing chronic pain states associated with neuropathic pain.

## 7. Preclinical Evidence and Mechanisms of GLP-1RA-Based Therapies for Neuropathic Pain: Clinical Trials, Diabetic Neuropathy, and Combination Therapies

Several animal studies have demonstrated the potential of GLP-1RAs in alleviating neuropathic pain. For instance, in a rat model of sciatic nerve injury, treatment with exenatide reduced pain behaviors, including hyperalgesia and allodynia [106]. This suggests that GLP-1RAs could modulate both peripheral and central pain mechanisms. The described analgesic effects in these studies might result from the anti-inflammatory, antioxidative, and neuroprotective effects of GLP-1RAs that together decrease pain, as described above. Furthermore, the neurogenic effect of GLP-1RAs may help in the neuronal repair and regeneration of nerves in damaged tissues.

Although clinical evidence is still limited, some trials have explored the effects of GLP-1RA-based therapies on pain management in patients with conditions such as diabetic neuropathy. A clinical trial evaluated the effect of liraglutide on body weight and pain in patients with overweight and knee osteoarthritis that explored the effects of liraglutide and discovered a significant decrease in pain scores as well as an improvement in quality-of-life scores, suggesting the potential therapeutic role of GLP-1RAs in pain treatment [19,107]. In clinical settings, GLP-1RAs are often combined with other therapies for diabetes management [108]. However, the synergistic effects of GLP-1RA-based therapies with traditional pain medications (such as gabapentinoids or opioids) could be explored in future trials to better assess their role in comprehensive neuropathic pain treatment regimens. A summary of the key preclinical and clinical findings on GLP-1RAs and DPP-4is in neuropathic pain models is presented in Table 1, highlighting their proposed mechanisms of action and therapeutic outcomes across various studies.

Although GLP-1-RA-based therapies have shown great promise as treatments for neuropathic pain, they may be more efficient in combination with other pain relievers. Combining GLP-1RAs with existing treatments for neuropathic pain, such as gabapentinoids, opioids, or non-steroidal anti-inflammatory drugs (NSAIDs), could offer synergistic effects.

### 7.1. Synergy with Gabapentinoids and Opioids

Many patients use gabapentin and pregabalin to help manage their neuropathic pain. Combining these drugs with GLP-1RAs can enhance the relief of pain through targeting multiple mechanisms, including reducing inflammation, oxidative stress, and central sensitization. Preliminary studies indicate that combining GLP-1RAs with opioids may lead to a decrease in the dose of opioids necessary, thereby decreasing the risk of opioid addiction and side effects [109].

### 7.2. Benefits in Diabetic Neuropathy

Unlike its conventional treatment, pregabalin, GLP-1-RA-based therapies can aid in better glycemic control in patients sustaining diabetic neuropathy [109]. The therapies provide a combination of treating the metabolic changes while lowering the severity of the pain pathways for dual relief. For patients of neuropathic pain or neuropathy, this could be really beneficial.

**Table 1:** Comparison of preclinical and clinical findings on GLP-1RAs and DPP-4is in neuropathic pain.

Aspect	GLP-1 Receptor Agonists (GLP-1RAs)	DPP-4 Inhibitors (DPP-4is)
Mechanism of Action	Activation of GLP-1 receptor → modulates insulin, inflammation, oxidative stress	Inhibits degradation of GLP-1 → indirectly enhances GLP-1 signaling
Preclinical Findings	→ Reduces pro-inflammatory cytokines (e.g., *TNF*-α, IL-1β) [25] → Lowers oxidative stress markers (e.g., MDA, 8-OHdG) [110] → Restores mitochondrial function [92] → Decreases glial cell activation and neuroinflammation [92] → Improves pain behaviors in diabetic neuropathy and nerve injury models [111]	→ Reduces neuroinflammation via microglial and astrocyte inhibition [11,47] → Enhances neuronal survival [112] → Lowers ROS levels and improves mitochondrial integrity [31] → Shows antinociceptive effects in sciatic nerve injury models [11]
Clinical Findings	→ Small-scale studies show reduced peripheral neuropathy symptoms [18]. → Improvements in quality-of-life scores in diabetic and osteoarthritis patients [113,114] → Ongoing trials suggest possible central modulation of pain pathways [10]	→ Reduction in systemic inflammatory markers (e.g., CRP, IL-6) in T2DM patients [115] → Limited direct clinical data in neuropathic pain, but systemic anti-inflammatory effect is promising
CNS Penetration	→ Crosses blood–brain barrier (e.g., liraglutide, semaglutide) → potential central effects [116]	→ Limited CNS penetration; indirect CNS effects through peripheral GLP-1 elevation [117]
Key Molecular Targets	→ GLP-1 receptor in CNS and peripheral nerves; AMPK, PGC-1α (mitochondrial pathways) [8]	→ DPP-4 enzyme on neurons/glial cells; prolongs endogenous GLP-1 action [118]
Synergistic Potential	→ Shown benefit in combination with gabapentinoids or opioids to reduce dosage needs [119,120]	→ May enhance effects when co-administered with opioids; mitigates morphine tolerance via glial inhibition [12]
Therapeutic Implications	→ Dual benefits: pain modulation and metabolic control; potential for CNS-focused drug development	→ May be safer alternatives to NSAIDs or opioids; promising for diabetic patients with comorbid pain
Limitations/Challenges	→ Debate on GLP-1R expression in spinal cord; need for targeted delivery for CNS	→ Lack of clinical studies in neuropathic pain; lower potency compared to GLP-1RAs

## 8. Future Directions and Challenges in Translating GLP-1RA-Based Therapies to Neuropathic Pain Treatment

One of the key challenges in advancing GLP-1RA-based therapies for neuropathic pain is the unclear expression of GLP-1R in the spinal cord. While some studies suggest the presence of GLP-1R in spinal cord neurons, others have reported contrary results. Further research using advanced techniques, such as single-cell RNA sequencing, may help clarify the expression patterns of GLP-1R in the spinal cord and determine its importance in neuropathic pain. While GLP-1RAs have promising antinociceptive effects in neuropathic pain, their therapeutic potential in pain management may require optimization of their delivery. The BBB represents a major challenge; however, new formulations or delivery methods, such as intrathecal or transdermal delivery systems, could vastly enhance the delivery of GLP-1RAs to sites of action in targeting the CNS and spinal cord.

As GLP-1RAs are primarily used for diabetes, long-term safety data in neuropathic pain patients are needed. Although GLP-1RAs have a favorable safety profile in diabetes management, their long-term use in non-diabetic neuropathic pain populations requires further evaluation.

## 9. Conclusions

GLP-1RAs and DPP-4is have demonstrated significant promise in managing neuropathic pain through their different mechanisms. By targeting inflammation, oxidative stress, mitochondrial dysfunction, and central pain processing, they have emerged as a new therapeutic approach for neuropathic pain management. While preclinical data are promising, more clinical trials are needed to assess their efficacy and safety in diverse neuropathic pain populations. Future research should also focus on optimizing GLP-1RAs for CNS targeting, resolving inconsistencies in spinal cord expression, and exploring combination therapies for enhanced pain relief.

In addition, translating these findings into clinical practice presents several challenges. Large-scale, randomized clinical trials are essential to establish therapeutic efficacy and define optimal dosing strategies. Long-term safety evaluations are also critical, particularly in populations with chronic neuropathic pain conditions. Addressing these research gaps will be key to fully realizing the therapeutic potential of GLP-1-based interventions in neuropathic pain management.

## Data Availability

No new data were created or analyzed in this study.

## References

[1] Colloca L., Ludman T., Bouhassira D., Baron R., Dickenson A.H., Yarnitsky D., Freeman R., Truini A., Attal N., Finnerup N.B. (2017). Neuropathic pain. Nat. Rev. Dis. Primers.

[2] Leoni M.L.G., Mercieri M., Viswanath O., Cascella M., Rekatsina M., Pasqualucci A., Caruso A., Varrassi G. (2025). Neuropathic Pain: A Comprehensive Bibliometric Analysis of Research Trends, Contributions, and Future Directions. Curr. Pain Headache Rep..

[3] Meacham K., Shepherd A., Mohapatra D.P., Haroutounian S. (2017). Neuropathic Pain: Central vs. Peripheral Mechanisms. Curr. Pain Headache Rep..

[4] Campbell J.N., Meyer R.A. (2006). Mechanisms of neuropathic pain. Neuron.

[5] Nashtahosseini Z., Eslami M., Paraandavaji E., Haraj A., Dowlat B.F., Hosseinzadeh E., Oksenych V., Naderian R. (2025). Cytokine Signaling in Diabetic Neuropathy: A Key Player in Peripheral Nerve Damage. Biomedicines.

[6] Gong N., Xiao Q., Zhu B., Zhang C.Y., Wang Y.C., Fan H., Ma A.N., Wang Y.X. (2014). Activation of spinal glucagon-like peptide-1 receptors specifically suppresses pain hypersensitivity. J. Neurosci. Off. J. Soc. Neurosci..

[7] Shekunova E.V., Kashkin V.A., Muzhikyan A.А., Makarova M.N., Balabanyan V.Y., Makarov V.G. (2020). Therapeutic efficacy of arginine-rich exenatide on diabetic neuropathy in rats. Eur. J. Pharmacol..

[8] Zheng Z., Zong Y., Ma Y., Tian Y., Pang Y., Zhang C., Gao J. (2024). Glucagon-like peptide-1 receptor: Mechanisms and advances in therapy. Signal Transduct. Target. Ther..

[9] Gallwitz B. (2019). Clinical Use of DPP-4 Inhibitors. Front. Endocrinol..

[10] He Y., Xu B., Zhang M., Chen D., Wu S., Gao J., Liu Y., Zhang Z., Kuang J., Fang Q. (2025). Advances in GLP-1 receptor agonists for pain treatment and their future potential. J. Headache Pain.

[11] Kuthati Y., Rao V.N., Busa P., Wong C.S. (2021). Teneligliptin Exerts Antinociceptive Effects in Rat Model of Partial Sciatic Nerve Transection Induced Neuropathic Pain. Antioxidants.

[12] Kuthati Y., Rao V.N., Huang W.H., Busa P., Wong C.S. (2023). Teneligliptin Co-Infusion Alleviates Morphine Tolerance by Inhibition of Spinal Microglial Cell Activation in Streptozotocin-Induced Diabetic Rats. Antioxidants.

[13] Lee S.-O., Kuthati Y., Huang W.-H., Wong C.-S. (2024). Semaglutide Ameliorates Diabetic Neuropathic Pain by Inhibiting Neuroinflammation in the Spinal Cord. Cells.

[14] Olukorode J.O., Orimoloye D.A., Nwachukwu N.O., Onwuzo C.N., Oloyede P.O., Fayemi T., Odunaike O.S., Ayobami-Ojo P.S., Divine N., Alo D.J. (2024). Recent Advances and Therapeutic Benefits of Glucagon-Like Peptide-1 (GLP-1) Agonists in the Management of Type 2 Diabetes and Associated Metabolic Disorders. Cureus.

[15] Zhao X., Wang M., Wen Z., Lu Z., Cui L., Fu C., Xue H., Liu Y., Zhang Y. (2021). GLP-1 Receptor Agonists: Beyond Their Pancreatic Effects. Front. Endocrinol..

[16] Alharbi S.H. (2024). Anti-inflammatory role of glucagon-like peptide 1 receptor agonists and its clinical implications. Ther. Adv. Endocrinol. Metab..

[17] Daniels S., Karlsson C., Schrauwen P., Parker V.E.R. (2025). Glucagon-like peptide-1 receptor agonism and end-organ protection. Trends Endocrinol. Metab..

[18] Dhanapalaratnam R., Issar T., Poynten A.M., Milner K.L., Kwai N.C.G., Krishnan A.V. (2025). Impact of glucagon-like peptide-1 receptor agonists on axonal function in diabetic peripheral neuropathy. J. Neurophysiol..

[19] Halloum W., Dughem Y.A., Beier D., Pellesi L. (2024). Glucagon-like peptide-1 (GLP-1) receptor agonists for headache and pain disorders: A systematic review. J. Headache Pain.

[20] Serrano Afonso A., Carnaval T., Videla Cés S. (2021). Combination Therapy for Neuropathic Pain: A Review of Recent Evidence. J. Clin. Med..

[21] Lindborg J.A., Niemi J.P., Howarth M.A., Liu K.W., Moore C.Z., Mahajan D., Zigmond R.E. (2018). Molecular and cellular identification of the immune response in peripheral ganglia following nerve injury. J. Neuroinflammation.

[22] Smith J.A., Das A., Ray S.K., Banik N.L. (2012). Role of pro-inflammatory cytokines released from microglia in neurodegenerative diseases. Brain Res. Bull..

[23] Breitinger U., Breitinger H.G. (2023). Excitatory and inhibitory neuronal signaling in inflammatory and diabetic neuropathic pain. Mol. Med..

[24] Houldsworth A. (2024). Role of oxidative stress in neurodegenerative disorders: A review of reactive oxygen species and prevention by antioxidants. Brain Commun..

[25] Wang L., Li X., Men X., Liu X., Luo J. (2025). Research progress on antioxidants and protein aggregation inhibitors in cataract prevention and therapy (Review). Mol. Med. Rep..

[26] Carrasco C., Naziroǧlu M., Rodríguez A.B., Pariente J.A. (2018). Neuropathic Pain: Delving into the Oxidative Origin and the Possible Implication of Transient Receptor Potential Channels. Front. Physiol..

[27] Espinoza N., Papadopoulos V. (2025). Role of Mitochondrial Dysfunction in Neuropathy. Int. J. Mol. Sci..

[28] Gaggini M., Sabatino L., Suman A.F., Chatzianagnostou K., Vassalle C. (2025). Insights into the Roles of GLP-1, DPP-4, and SGLT2 at the Crossroads of Cardiovascular, Renal, and Metabolic Pathophysiology. Cells.

[29] Kopp K.O., Glotfelty E.J., Li Y., Greig N.H. (2022). Glucagon-like peptide-1 (GLP-1) receptor agonists and neuroinflammation: Implications for neurodegenerative disease treatment. Pharmacol. Res..

[30] Deacon C.F. (2019). Physiology and Pharmacology of DPP-4 in Glucose Homeostasis and the Treatment of Type 2 Diabetes. Front. Endocrinol..

[31] Shao S., Xu Q., Yu X., Pan R., Chen Y. (2020). Dipeptidyl peptidase 4 inhibitors and their potential immune modulatory functions. Pharmacol. Ther..

[32] Monti G., Gomes Moreira D., Richner M., Mutsaers H.A.M., Ferreira N., Jan A. (2022). GLP-1 Receptor Agonists in Neurodegeneration: Neurovascular Unit in the Spotlight. Cells.

[33] Zeng N., Cutts E.J., Lopez C.B., Kaur S., Duran M., Virkus S.A., Hardaway J.A. (2021). Anatomical and Functional Characterization of Central Amygdala Glucagon-Like Peptide 1 Receptor Expressing Neurons. Front. Behav. Neurosci..

[34] Graham D.L., Durai H.H., Trammell T.S., Noble B.L., Mortlock D.P., Galli A., Stanwood G.D. (2020). A novel mouse model of glucagon-like peptide-1 receptor expression: A look at the brain. J. Comp. Neurol..

[35] Kanoski S.E., Fortin S.M., Arnold M., Grill H.J., Hayes M.R. (2011). Peripheral and central GLP-1 receptor populations mediate the anorectic effects of peripherally administered GLP-1 receptor agonists, liraglutide and exendin-4. Endocrinology.

[36] Jensen C.B., Pyke C., Rasch M.G., Dahl A.B., Knudsen L.B., Secher A. (2017). Characterization of the Glucagonlike Peptide-1 Receptor in Male Mouse Brain Using a Novel Antibody and In Situ Hybridization. Endocrinology.

[37] Vahl T.P., Tauchi M., Durler T.S., Elfers E.E., Fernandes T.M., Bitner R.D., Ellis K.S., Woods S.C., Seeley R.J., Herman J.P. (2007). Glucagon-Like Peptide-1 (GLP-1) Receptors Expressed on Nerve Terminals in the Portal Vein Mediate the Effects of Endogenous GLP-1 on Glucose Tolerance in Rats. Endocrinology.

[38] Timper K., Del Río-Martín A., Cremer A.L., Bremser S., Alber J., Giavalisco P., Varela L., Heilinger C., Nolte H., Trifunovic A. (2020). GLP-1 Receptor Signaling in Astrocytes Regulates Fatty Acid Oxidation, Mitochondrial Integrity, and Function. Cell Metab..

[39] Hunter K., Hölscher C. (2012). Drugs developed to treat diabetes, liraglutide and lixisenatide, cross the blood brain barrier and enhance neurogenesis. BMC Neurosci..

[40] Athauda D., Foltynie T. (2016). The glucagon-like peptide 1 (GLP) receptor as a therapeutic target in Parkinson’s disease: Mechanisms of action. Drug Discov. Today.

[41] Baggio L.L., Drucker D.J. (2014). Glucagon-like peptide-1 receptors in the brain: Controlling food intake and body weight. J. Clin. Invest..

[42] Sisley S., Gutierrez-Aguilar R., Scott M., D’Alessio D.A., Sandoval D.A., Seeley R.J. (2014). Neuronal GLP1R mediates liraglutide’s anorectic but not glucose-lowering effect. J. Clin. Invest..

[43] Li Y., Chigurupati S., Holloway H.W., Mughal M., Tweedie D., Bruestle D.A., Mattson M.P., Wang Y., Harvey B.K., Ray B. (2012). Exendin-4 Ameliorates Motor Neuron Degeneration in Cellular and Animal Models of Amyotrophic Lateral Sclerosis. PLoS ONE.

[44] Chen B., Yu X., Horvath-Diano C., Ortuño M.J., Tschöp M.H., Jastreboff A.M., Schneeberger M. (2024). GLP-1 programs the neurovascular landscape. Cell Metab..

[45] Tong G., Peng T., Chen Y., Sha L., Dai H., Xiang Y., Zou Z., He H., Wang S. (2022). Effects of GLP-1 Receptor Agonists on Biological Behavior of Colorectal Cancer Cells by Regulating PI3K/AKT/mTOR Signaling Pathway. Front. Pharmacol..

[46] Rosenstock J., Zinman B. (2007). Dipeptidyl peptidase-4 inhibitors and the management of type 2 diabetes mellitus. Curr. Opin. Endocrinol. Diabetes Obes..

[47] Király K., Kozsurek M., Lukácsi E., Barta B., Alpár A., Balázsa T., Fekete C., Szabon J., Helyes Z., Bölcskei K. (2018). Glial cell type-specific changes in spinal dipeptidyl peptidase 4 expression and effects of its inhibitors in inflammatory and neuropatic pain. Sci. Rep..

[48] McKillop A.M., Stevenson C.L., Moran B.M., Abdel-Wahab Y.H.A., Flatt P.R. (2018). Tissue expression of DPP-IV in obesity-diabetes and modulatory effects on peptide regulation of insulin secretion. Peptides.

[49] Deacon C.F. (2004). Circulation and degradation of GIP and GLP-1. Horm. Metab. Res..

[50] Craddy P., Palin H.J., Johnson K.I. (2014). Comparative effectiveness of dipeptidylpeptidase-4 inhibitors in type 2 diabetes: A systematic review and mixed treatment comparison. Diabetes Ther..

[51] Madsbad S. (2009). Exenatide and liraglutide: Different approaches to develop GLP-1 receptor agonists (incretin mimetics)--preclinical and clinical results. Best Pract. Res. Clin. Endocrinol. Metab..

[52] Gerich J. (2010). DPP-4 inhibitors: What may be the clinical differentiators?. Diabetes Res. Clin. Pr..

[53] Stonehouse A., Walsh B., Cuddihy R. (2011). Exenatide once-weekly clinical development: Safety and efficacy across a range of background therapies. Diabetes Technol. Ther..

[54] Baggio L.L., Drucker D.J. (2007). Biology of incretins: GLP-1 and GIP. Gastroenterology.

[55] Seufert J., Gallwitz B. (2014). The extra-pancreatic effects of GLP-1 receptor agonists: A focus on the cardiovascular, gastrointestinal and central nervous systems. Diabetes Obes. Metab..

[56] Cifuentes L., Camilleri M., Acosta A. (2021). Gastric Sensory and Motor Functions and Energy Intake in Health and Obesity-Therapeutic Implications. Nutrients.

[57] Maselli D.B., Camilleri M. (2021). Effects of GLP-1 and Its Analogs on Gastric Physiology in Diabetes Mellitus and Obesity. Adv. Exp. Med. Biol..

[58] Uccellatore A., Genovese S., Dicembrini I., Mannucci E., Ceriello A. (2015). Comparison Review of Short-Acting and Long-Acting Glucagon-like Peptide-1 Receptor Agonists. Diabetes Ther. Res. Treat. Educ. Diabetes Relat. Disord..

[59] Gilbert M.P., Pratley R.E. (2020). GLP-1 Analogs and DPP-4 Inhibitors in Type 2 Diabetes Therapy: Review of Head-to-Head Clinical Trials. Front. Endocrinol..

[60] Nauck M.A., Quast D.R., Wefers J., Meier J.J. (2021). GLP-1 receptor agonists in the treatment of type 2 diabetes—State-of-the-art. Mol. Metab..

[61] Latif W., Lambrinos K.J., Patel P., Rodriguez R. (2025). Compare and Contrast the Glucagon-Like Peptide-1 Receptor Agonists (GLP1RAs). StatPearls.

[62] Liu C., Wu T., Ren N. (2023). Glucagon-like peptide-1 receptor agonists for the management of diabetic peripheral neuropathy. Front. Endocrinol..

[63] Catalisano G., Campione G.M., Spurio G., Galvano A.N., di Villalba C.P., Giarratano A., Alongi A., Ippolito M., Cortegiani A. (2024). Neuropathic pain, antidepressant drugs, and inflammation: A narrative review. J. Anesth. Analg. Crit. Care.

[64] Meurot C., Martin C., Sudre L., Breton J., Bougault C., Rattenbach R., Bismuth K., Jacques C., Berenbaum F. (2022). Liraglutide, a glucagon-like peptide 1 receptor agonist, exerts analgesic, anti-inflammatory and anti-degradative actions in osteoarthritis. Sci. Rep..

[65] Zhang Q., Li Q., Liu S., Zheng H., Ji L., Yi N., Bao W., Zhu X., Sun W., Liu X. (2022). Glucagon-like peptide-1 receptor agonist attenuates diabetic neuropathic pain via inhibition of NOD-like receptor protein 3 inflammasome in brain microglia. Diabetes Res. Clin. Pract..

[66] Xu M., Wu H.Y., Liu H., Gong N., Wang Y.R., Wang Y.X. (2017). Morroniside, a secoiridoid glycoside from Cornus officinalis, attenuates neuropathic pain by activation of spinal glucagon-like peptide-1 receptors. Br. J. Pharmacol..

[67] Yang X., Feng P., Zhang X., Li D., Wang R., Ji C., Li G., Hölscher C. (2019). The diabetes drug semaglutide reduces infarct size, inflammation, and apoptosis, and normalizes neurogenesis in a rat model of stroke. Neuropharmacology.

[68] Ma L., Ju P., Wang W., Wei J., Wang W., Zhao M., Ahmad K.A., Wang Y., Chen J. (2021). Microglial Activation of GLP-1R Signaling in Neuropathic Pain Promotes Gene Expression Adaption Involved in Inflammatory Responses. Neural Plast..

[69] Hendarto H., Inoguchi T., Maeda Y., Ikeda N., Zheng J., Takei R., Yokomizo H., Hirata E., Sonoda N., Takayanagi R. (2012). GLP-1 analog liraglutide protects against oxidative stress and albuminuria in streptozotocin-induced diabetic rats via protein kinase A-mediated inhibition of renal NAD(P)H oxidases. Metab. Clin. Exp..

[70] Ma L., Peng S., Wei J., Zhao M., Ahmad K.A., Chen J., Wang Y.X. (2021). Spinal microglial β-endorphin signaling mediates IL-10 and exenatide-induced inhibition of synaptic plasticity in neuropathic pain. CNS Neurosci. Ther..

[71] Mokhtari T., Lu M., El-Kenawy A.E.-M. (2023). Potential anxiolytic and antidepressant-like effects of luteolin in a chronic constriction injury rat model of neuropathic pain: Role of oxidative stress, neurotrophins, and inflammatory factors. Int. Immunopharmacol..

[72] Zhang G.L., Liu Y., Liu Y.F., Huang X.T., Tao Y., Chen Z.H., Lai H.L. (2024). Teneligliptin mitigates diabetic cardiomyopathy by inhibiting activation of the NLRP3 inflammasome. World J. Diabetes.

[73] Khan T., Waseem R., Zehra Z., Aiman A., Bhardwaj P., Ansari J., Hassan M.I., Islam A. (2022). Mitochondrial Dysfunction: Pathophysiology and Mitochondria-Targeted Drug Delivery Approaches. Pharmaceutics.

[74] Pan X., Xu S., Li J., Tong N. (2020). The Effects of DPP-4 Inhibitors, GLP-1RAs, and SGLT-2/1 Inhibitors on Heart Failure Outcomes in Diabetic Patients With and Without Heart Failure History: Insights From CVOTs and Drug Mechanism. Front. Endocrinol..

[75] Mehdi S.F., Pusapati S., Anwar M.S., Lohana D., Kumar P., Nandula S.A., Nawaz F.K., Tracey K., Yang H., LeRoith D. (2023). Glucagon-like peptide-1: A multi-faceted anti-inflammatory agent. Front. Immunol..

[76] Moustafa P.E., Abdelkader N.F., El Awdan S.A., El-Shabrawy O.A., Zaki H.F. (2018). Liraglutide ameliorated peripheral neuropathy in diabetic rats: Involvement of oxidative stress, inflammation and extracellular matrix remodeling. J. Neurochem..

[77] Noguchi T., Katoh H., Nomura S., Okada K., Watanabe M. (2024). The GLP-1 receptor agonist exenatide improves recovery from spinal cord injury by inducing macrophage polarization toward the M2 phenotype. Front. Neurosci..

[78] Lambadiari V., Thymis J., Kouretas D., Skaperda Z., Tekos F., Kousathana F., Kountouri A., Balampanis K., Parissis J., Andreadou I. (2021). Effects of a 12-Month Treatment with Glucagon-like Peptide-1 Receptor Agonists, Sodium-Glucose Cotransporter-2 Inhibitors, and Their Combination on Oxidant and Antioxidant Biomarkers in Patients with Type 2 Diabetes. Antioxidants.

[79] Zhang T.J., Qiu Y., Hua Z. (2019). The Emerging Perspective of Morphine Tolerance: MicroRNAs. Pain Res. Manag..

[80] Satoh-Asahara N., Sasaki Y., Wada H., Tochiya M., Iguchi A., Nakagawachi R., Odori S., Kono S., Hasegawa K., Shimatsu A. (2013). A dipeptidyl peptidase-4 inhibitor, sitagliptin, exerts anti-inflammatory effects in type 2 diabetic patients. Metab. Clin. Exp..

[81] Xie W., Song X., Liu Z. (2018). Impact of dipeptidyl-peptidase 4 inhibitors on cardiovascular diseases. Vasc. Pharmacol..

[82] Feng Y., Shang B., Yang Y., Zhang D., Liu C., Qin Z., Zhou Y., Meng J., Liu X. (2025). Impact of DPP-4 Inhibitors on Interleukin Levels in Type 2 Diabetes Mellitus. J. Clin. Endocrinol. Metab..

[83] Nesto R. (2004). C-reactive protein, its role in inflammation, Type 2 diabetes and cardiovascular disease, and the effects of insulin-sensitizing treatment with thiazolidinediones. Diabet. Med. A J. Br. Diabet. Assoc..

[84] Ghasemi A., Jalali Kondori B., Ghasemi M., Bahari Z. (2023). Potential Role of Oxidative Stress on the Pathophysiology of Neuropathic Pain in the Inflammatory Diseases. J. Adv. Med. Biomed. Res..

[85] Teixeira-Santos L., Albino-Teixeira A., Pinho D. (2020). Neuroinflammation, oxidative stress and their interplay in neuropathic pain: Focus on specialized pro-resolving mediators and NADPH oxidase inhibitors as potential therapeutic strategies. Pharmacol. Res..

[86] Oh Y.S., Jun H.S. (2017). Effects of Glucagon-Like Peptide-1 on Oxidative Stress and Nrf2 Signaling. Int. J. Mol. Sci..

[87] Kabahizi A., Wallace B., Lieu L., Chau D., Dong Y., Hwang E.S., Williams K.W. (2022). Glucagon-like peptide-1 (GLP-1) signalling in the brain: From neural circuits and metabolism to therapeutics. Br. J. Pharmacol..

[88] Lin Q., Li K., Chen Y., Xie J., Wu C., Cui C., Deng B. (2023). Oxidative Stress in Diabetic Peripheral Neuropathy: Pathway and Mechanism-Based Treatment. Mol. Neurobiol..

[89] Eftekharpour E., Fernyhough P. (2022). Oxidative Stress and Mitochondrial Dysfunction Associated with Peripheral Neuropathy in Type 1 Diabetes. Antioxid. Redox Signal..

[90] Ghosh P., Fontanella R.A., Scisciola L., Pesapane A., Taktaz F., Franzese M., Puocci A., Ceriello A., Prattichizzo F., Rizzo M.R. (2023). Targeting redox imbalance in neurodegeneration: Characterizing the role of GLP-1 receptor agonists. Theranostics.

[91] Chidambaram S.B., Anand N., Varma S.R., Ramamurthy S., Vichitra C., Sharma A., Mahalakshmi A.M., Essa M.M. (2024). Superoxide dismutase and neurological disorders. IBRO Neurosci. Rep..

[92] Luna-Marco C., de Marañon A.M., Hermo-Argibay A., Rodriguez-Hernandez Y., Hermenejildo J., Fernandez-Reyes M., Apostolova N., Vila J., Sola E., Morillas C. (2023). Effects of GLP-1 receptor agonists on mitochondrial function, inflammatory markers and leukocyte-endothelium interactions in type 2 diabetes. Redox Biol..

[93] Wu Q., Li D., Huang C., Zhang G., Wang Z., Liu J., Yu H., Song B., Zhang N., Li B. (2022). Glucose control independent mechanisms involved in the cardiovascular benefits of glucagon-like peptide-1 receptor agonists. Biomed. Pharmacother..

[94] Zong Y., Li H., Liao P., Chen L., Pan Y., Zheng Y., Zhang C., Liu D., Zheng M., Gao J. (2024). Mitochondrial dysfunction: Mechanisms and advances in therapy. Signal Transduct. Target. Ther..

[95] Yang C., Zhao X., An X., Zhang Y., Sun W., Zhang Y., Duan Y., Kang X., Sun Y., Jiang L. (2023). Axonal transport deficits in the pathogenesis of diabetic peripheral neuropathy. Front. Endocrinol..

[96] Westermeier F., Fisman E.Z. (2025). Glucagon-like peptide-1 receptor agonists (GLP-1RAs) and cardiometabolic protection: Historical development and future challenges. Cardiovasc. Diabetol..

[97] Yip J.M.X., Chiang G.S.H., Lee I.C.J., Lehming-Teo R., Dai K., Dongol L., Wang L.Y.-T., Teo D., Seah G.T., Lehming N. (2025). Mitochondria and the Repurposing of Diabetes Drugs for Off-Label Health Benefits. Int. J. Mol. Sci..

[98] Wang X., Wang L. (2025). AMPK-Mediated Multi-Organ Protective Effects of GLP-1 Receptor Agonists. Health Metab..

[99] Hong C.T., Chen J.H., Hu C.J. (2024). Role of glucagon-like peptide-1 receptor agonists in Alzheimer’s disease and Parkinson’s disease. J. Biomed. Sci..

[100] Go E.J., Hwang S.-M., Jo H., Rahman M.M., Park J., Lee J.Y., Jo Y.Y., Lee B.-G., Jung Y., Berta T. (2024). GLP-1 and its derived peptides mediate pain relief through direct TRPV1 inhibition without affecting thermoregulation. Exp. Mol. Med..

[101] Diz-Chaves Y., Mastoor Z., Spuch C., González-Matías L.C., Mallo F. (2022). Anti-Inflammatory Effects of GLP-1 Receptor Activation in the Brain in Neurodegenerative Diseases. Int. J. Mol. Sci..

[102] Gabery S., Salinas C.G., Paulsen S.J., Ahnfelt-Rønne J., Alanentalo T., Baquero A.F., Buckley S.T., Farkas E., Fekete C., Frederiksen K.S. (2020). Semaglutide lowers body weight in rodents via distributed neural pathways. JCI Insight.

[103] Cabou C., Burcelin R. (2011). GLP-1, the gut-brain, and brain-periphery axes. Rev. Diabet. Stud. RDS.

[104] Wen Z., Sun W., Wang H., Chang R., Wang J., Song C., Zhang S., Ni Q., An X. (2025). Comparison of the effectiveness and safety of GLP-1 receptor agonists for type 2 diabetes mellitus patients with overweight/obesity: A systematic review and network meta-analysis. Diabetes Res. Clin. Pract..

[105] Latremoliere A., Woolf C.J. (2009). Central sensitization: A generator of pain hypersensitivity by central neural plasticity. J. Pain.

[106] Fujita S., Ushio S., Ozawa N., Masuguchi K., Kawashiri T., Oishi R., Egashira N. (2015). Exenatide Facilitates Recovery from Oxaliplatin-Induced Peripheral Neuropathy in Rats. PLoS ONE.

[107] Gudbergsen H., Henriksen M., Wæhrens E.E., Overgaard A., Bliddal H., Christensen R., Boesen M.P., Knop F.K.K., Astrup A., Rasmussen M.U. (2019). Effect of liraglutide on body weight and pain in patients with overweight and knee osteoarthritis: Protocol for a randomised, double-blind, placebo-controlled, parallel-group, single-centre trial. BMJ Open.

[108] Collins L., Costello R.A. (2025). Glucagon-Like Peptide-1 Receptor Agonists. StatPearls.

[109] Qeadan F., McCunn A., Tingey B. (2025). The association between glucose-dependent insulinotropic polypeptide and/or glucagon-like peptide-1 receptor agonist prescriptions and substance-related outcomes in patients with opioid and alcohol use disorders: A real-world data analysis. Addiction.

[110] Birnbaum Y. (2025). Glucagon-Like Peptide-1 Receptor Agonists for Abdominal Aortic Aneurysm?. Cardiovasc. Drugs Ther..

[111] García-Casares N., González-González G., de la Cruz-Cosme C., Garzón-Maldonado F.J., de Rojas-Leal C., Ariza M.J., Narváez M., Barbancho M., García-Arnés J.A., Tinahones F.J. (2023). Effects of GLP-1 receptor agonists on neurological complications of diabetes. Rev. Endocr. Metab. Disord..

[112] Jiang X., Li J., Yao X., Ding H., Gu A., Zhou Z. (2024). Neuroprotective effects of dipeptidyl peptidase 4 inhibitor on Alzheimer’s disease: A narrative review. Front. Pharmacol..

[113] Meurot C., Jacques C., Martin C., Sudre L., Breton J., Rattenbach R., Bismuth K., Berenbaum F. (2022). Targeting the GLP-1/GLP-1R axis to treat osteoarthritis: A new opportunity?. J. Orthop. Transl..

[114] Lopes A.C., Lourenço O., Morgado M. (2024). SGLT2i and GLP1RA effects in patients followed in a hospital diabetology consultation. Expert Rev. Clin. Pharmacol..

[115] Baggio L.L., Varin E.M., Koehler J.A., Cao X., Lokhnygina Y., Stevens S.R., Holman R.R., Drucker D.J. (2020). Plasma levels of DPP4 activity and sDPP4 are dissociated from inflammation in mice and humans. Nat. Commun..

[116] Dong M., Wen S., Zhou L. (2022). The Relationship Between the Blood-Brain-Barrier and the Central Effects of Glucagon-Like Peptide-1 Receptor Agonists and Sodium-Glucose Cotransporter-2 Inhibitors. Diabetes Metab. Syndr. Obes. Targets Ther..

[117] Darsalia V., Johansen O.E., Lietzau G., Nyström T., Klein T., Patrone C. (2019). Dipeptidyl Peptidase-4 Inhibitors for the Potential Treatment of Brain Disorders; A Mini-Review With Special Focus on Linagliptin and Stroke. Front. Neurol..

[118] Al-Badri G., Leggio G.M., Musumeci G., Marzagalli R., Drago F., Castorina A. (2018). Tackling dipeptidyl peptidase IV in neurological disorders. Neural Regen. Res..

[119] Chuong V., Farokhnia M., Khom S., Pince C.L., Elvig S.K., Vlkolinsky R., Marchette R.C., Koob G.F., Roberto M., Vendruscolo L.F. (2023). The glucagon-like peptide-1 (GLP-1) analogue semaglutide reduces alcohol drinking and modulates central GABA neurotransmission. JCI Insight.

[120] Wang W., Volkow N.D., Wang Q., Berger N.A., Davis P.B., Kaelber D.C., Xu R. (2024). Semaglutide and Opioid Overdose Risk in Patients with Type 2 Diabetes and Opioid Use Disorder. JAMA Netw. Open.

